# Investigation of the Critical Biomass of Acclimated Microbial Communities to High Ammonia Concentrations for a Successful Bioaugmentation of Biogas Anaerobic Reactors with Ammonia Inhibition

**DOI:** 10.3390/microorganisms11071710

**Published:** 2023-06-30

**Authors:** Sotirios D. Kalamaras, Maria Lida Christou, Christos A. Tzenos, Sotirios Vasileiadis, Dimitrios G. Karpouzas, Thomas A. Kotsopoulos

**Affiliations:** 1 Department of Hydraulics, Soil Science and Agricultural Engineering, School of Agriculture, Aristotle University of Thessaloniki, 54124 Thessaloniki, Greece; christoum@agro.auth.gr (M.L.C.); catzenos@agro.auth.gr (C.A.T.); 2 Department of Biochemistry and Biotechnology, University of Thessaly, 41500 Larissa, Greece; sovasileiadis@uth.gr (S.V.); dkarpouzas@uth.gr (D.G.K.)

**Keywords:** anaerobic digestion, biogas, methane, bioaugmentation, critical biomass

## Abstract

This study aimed to investigate the role of the bioaugmented critical biomass that should be injected for successful bioaugmentation for addressing ammonia inhibition in anaerobic reactors used for biogas production. Cattle manure was used as a feedstock for anaerobic digestion (AD). A mixed microbial culture was acclimated to high concentrations of ammonia and used as a bioaugmented culture. Different volumes of bioaugmented culture were injected in batch anaerobic reactors under ammonia toxicity levels i.e., 4 g of NH_4_^+^-N L^−1^. The results showed that injecting a volume equal to 65.62% of the total working reactor volume yielded the best methane production. Specifically, this volume of bioaugmented culture resulted in methane production rates of 196.18 mL g^−1^ Volatile Solids (VS) and 245.88 mL g^−1^ VS after 30 and 60 days of AD, respectively. These rates were not significantly different from the control reactors (30d: 205.94 mL CH_4_ g^−1^ VS and 60d: 230.26 mL CH_4_ g^−1^ VS) operating without ammonia toxicity. Analysis of the microbial community using 16S rRNA gene sequencing revealed the dominance of acetoclastic methanogen members from the genus *Methanosaeta* in all reactors.

## 1. Introduction

Anaerobic digestion (AD) is a promising energy technology in which microorganisms degrade organic waste in the absence of oxygen to produce biogas. Biogas mainly consists of methane (CH_4_) and carbon dioxide (CO_2_) and it can be used as fuel in specific internal combustion engines for energy generation. AD is an environmentally friendly technology as it converts organic waste into a useful source of energy without releasing harmful greenhouse gases into the atmosphere. It also helps to reduce the volume of organic waste in landfills and has a crucial role in waste management in agriculture. AD has a favorable economic impact as the number of biogas plants, which utilize this process, is currently increasing, especially in European countries [1].

AD is a biological process that has limitations and problems related to various biological factors such as inhibition by toxic compounds. Ammonia is a common inhibitor compound in AD, generated by the degradation of nitrogen compounds contained in organic waste, such as proteins, urea, and nucleic acids [2,3]. In aqueous solutions, total ammonia nitrogen (TAN) is found in the form of ammonium ions (NH_4_^+^) and free ammonia (NH_3_), and their concentration is strongly dependent on the temperature and pH value of the solution. When the concentration of ammonia in an anaerobic reactor exceeds a certain level, it can negatively impact the activity and stability of the microbial community and especially of methanogenic Archaea [4,5]. Ammonia toxicity in anaerobic reactors is often expressed by lower biogas production and at high concentrations even with process failure. Thus, addressing ammonia inhibition is crucial for ensuring efficient and stable biogas production.

Bioaugmentation is a promising process to alleviate important problematic states of anaerobic reactors, including ammonia inhibition. It is a biological process that involves the addition of selected microbial cultures to enhance the performance of an existing microbial community in a biological system [6]. Bioaugmentation offers several advantages, such as the prompt and effective response of the added microbial community, ease of implementation, and economic feasibility [7]. The added microorganisms can improve the stability and productivity of the process by increasing the functional diversity and activity of the microbial community [8]. In the case of ammonia inhibition, bioaugmentation can help establish ammonia-tolerant microbial communities that can adapt to high ammonia concentrations and recover and maintain biogas production at levels comparable to those before ammonia inhibition [9].

Bioaugmentation can be performed by different types of microbial cultures-inoculum. Pure or mixed cultures are commonly used for tackling ammonia inhibition in anaerobic reactors. Pure cultures are single strains of microorganisms that are often well-characterized and have specific metabolic functions. Studies have shown successful bioaugmentation in anaerobic reactors with ammonia toxicity using pure cultures [10,11]. Moreover, a combination of pure cultures of a hydrogenotrophic methanogen (*Methanobrevibacter smithii*) and syntrophic acetate oxidizing bacteria (*Syntrophaceticu schinkii*) demonstrated the best results in methane production of ammonia-inhibited anaerobic reactors with a 71% increase compared to control, in a study that tested single and combinations of pure strain cultures [12]. However, using pure strains as bioaugmentation consortia incur significant expenses and pose particular technical obstacles such as the requirement for aseptic conditions and specialized nutrient media [13]. Alternatively, mixed culture inoculum can acclimate more efficiently to particular environmental conditions, such as ammonia toxicity, due to their functional diversity and synergistic interactions, resulting in increased resilience [14]. Successful bioaugmentation with mixed cultures has been reported to alleviate ammonia inhibition in AD by previous researchers [15,16,17]. Typically, mixed cultures used in bioaugmentation are obtained from anaerobic reactors that operate under similar conditions, including targeted concentration levels of ammonia toxicity. These mixed cultures are pre-acclimated to high ammonia conditions, making them more effective at degrading organic biomass under high ammonia concentrations. Analysis of the microbial community composition by DNA screening of the bioaugmentation mixed culture and the bioaugmented anaerobic reactors are performed to gain insight into the tested microbial communities [14]. By analyzing the DNA of different microbial communities, specific microorganisms could be selected and result in potentially optimized specific mixed cultures that could be capable of efficiently degrading organic waste and producing biogas in the presence of high levels of ammonia, thereby improving the stability and productivity of AD systems.

The effectiveness of bioaugmentation can be significantly influenced by the successful delivery of the bioaugmented culture inoculum [18]. Sustaining ammonia-tolerant microorganisms in anaerobic reactors can be challenging due to the competition from other microorganisms and the washout phenomenon, where microorganisms are washed out of the reactor due to high hydraulic retention times or low inoculum volume and density. Researchers have proposed various strategies for performing bioaugmentation in ammonia-inhibited anaerobic reactors, including different volumes, densities, and numbers of applications of the added microbial culture. Specifically, most of the researchers are using two [11,17,19] or multiple dosages [16,20,21,22] of bioaugmented culture throughout their experiments. However, finding the critical biomass and applying only one injection could save effort, time, and minimize costs. Therefore, determining the critical biomass of the bioaugmented culture is an important factor that can help maintain a stable microbial community in the reactor and enhance its performance under high ammonia concentrations [19]. To our knowledge, the investigation of the critical biomass of the bioaugmented culture to be injected into batch anaerobic reactors inhibited by ammonia for successful bioaugmentation, along with a simultaneous analysis of the adaptation of the bioaugmented culture in these reactors using 16S rRNA gene sequencing, has never been applied before.

This study aims to investigate the critical biomass required for successful bioaugmentation to address ammonia inhibition in anaerobic reactors that produce biogas. Acclimation of mixed microbial cultures to high ammonia concentrations was performed, to use them as bioaugmented cultures. Different volumes of bioaugmented culture were used to determine the critical biomass injection in batch anaerobic reactors. Methane potential was used to compare the effectiveness and the successful bioaugmentation of the different volumes of the bioaugmented microbial consortia. Microbial analysis based on 16S rRNA gene sequencing was performed to identify the composition of the bioaugmented culture. Moreover, samples were taken at the end of the experimental processes of AD to investigate the adaptation of the bioaugmented culture in the microbial community of the reactors.

## 2. Materials and Methods

### 2.1. Inoculum and Feedstock

Inoculum was obtained from a commercial biogas plant, Bioenergy Nigritas S.A. (Serres, Greece), located in Northern Greece, which operated under mesophilic temperature conditions (37 ± 1 °C). To eliminate any residual methane production, the inoculum was placed in an incubator at a constant temperature of 37 ± 0.3 °C for 5 days.

The feedstock for the anaerobic digestion (AD) experiment was fresh cattle manure sourced from a dairy cattle farm, also located in Northern Greece. Coarse materials were removed using a sieve, and the manure was stored at −20 °C after homogenization. Prior to introduction into the anaerobic reactors, the manure was kept at a temperature of 4 °C for 3 days. The characteristics of the inoculum and cattle manure are presented in Table 1.

To achieve high ammonia concentrations in the feedstock, we utilized ammonium chloride with a chemical purity of 99.99% (NH_4_Cl, Merck, Darmstadt, Germany).

### 2.2. Bioaugmentation Culture

A different inoculum obtained from a commercial biogas plant (Biogas Lagada S.A., Lagkadas, Greece) was used for the creation of the bioaugmented culture (Table 1). The bioaugmented culture resistant to high concentrations of ammonia was prepared by the inoculum and by gradually increasing ammonia concentrations in subsequent batch cultivation. Batch cultures were prepared in 1131.5 mL glass reactors with a starting working volume of 280 mL. The aforementioned cattle manure and D-glucose (C_6_H_12_O_6_·H_2_O, Duchefa Biochemie, Haarlem, The Netherlands) of purity greater than 99.50% were used as feedstock in a volumetric and volatile solids (2 g VS) ratio of 1:1. The bioaugmentation reactors were flushed with a gas mixture of N_2_ and CO_2_ (80/20% *v*/*v*) to carry out anaerobic conditions and placed inside an incubator operating at 37 ± 0.3 °C. Ammonium chloride was used to step increase the ammonia concentration during the creation of the bioaugmented culture as described previously [13]. The final ammonia concentration after 4 steps of increase was reached in terms of TAN and FAN at 5.20 ± 0.19 g L^−1^ and 254 ± 32 g L^−1^, respectively. The acclimated enrichment culture was 5× concentrated by centrifugation (10 min of 4500 rpm) in a refrigerated centrifuge (SL 8, Thermo Fisher Scientific, Dreieich, Germany). After centrifugation, the supernatant liquid was discarded, and the dense biomass that remained was used as concentrated bioaugmented culture. The characteristics of the bioaugmented culture are presented in Table 1.

### 2.3. Experimental Setup

Batch glass reactors were used with a total and working volume of 321 mL and 160 mL, respectively. The anaerobic conditions were achieved by flushing the reactors with a gas mixture of N_2_ and CO_2_ (80/20, *v*/*v*). The reactors were maintained under anaerobic conditions by closing them with rubber cups suitable for needle penetration during gas sampling acquisition. To investigate the critical biomass percentage at which bioaugmentation is achieved, seven different sets of reactors (in triplicate) with different contents were used and they are presented in Table 2. The PSA treatment was conducted to determine the potential production of the substrate with the addition of ammonia. The PS treatment was conducted to determine the maximum potential production of the substrate without the addition of ammonia. Two control treatments were conducted, IN to determine the potential production of the inoculum alone and PBIOA which contained bioaugmented culture with ammonia, to subtract accordingly the potential production from the bioaugmented treatments (BIOmin, BIOmed, and BIOmax), which was due to the bioaugmented culture. Three different volumes of bioaugmented culture were used to investigate the effectiveness of critical biomass in biogas production from anaerobic digestion inhibited by high ammonia concentrations. Bioaugmented culture was added to these different treatments named BIOmin, BIOmed, and BIOmax reactors in a percentage of approximately 21.87, 43.75, and 65.62% of the total working volume, respectively. Furthermore, inoculum, substrate, and ammonia were added to these reactors. Water was also added to all reactors to maintain the same working volume. All reactors were operated under mesophilic conditions (37 ± 0.3 °C).

### 2.4. Analytical Methods

Measurements of total Kjeldahl nitrogen (TKN), total ammonia (TAN), total solids (TS), and volatile solids (VS) were performed based on methods of APHA’s Standard Methods [23]. The pH value of solutions was measured with a bench tabletop digital pH meter (3520, Jenway, London, UK).

Biogas samples from the headspace of the reactors were collected using a gas-tight syringe that had a pressure lock and a needle attached to it. Throughout the initial 27 days of the experiment, daily biogas samples were collected from the reactors, followed by subsequent sampling intervals of 1 to 3 days until the 60th day from the start of the experiment. Methane content and production was monitored with a gas chromatograph (GC-2010plusAT, Shimadzu, Tokyo, Japan) equipped with a thermal conductivity detector with column characteristics and the method’s parameters described before [14]. Volatile fatty acids (VFAs) samples profile and concentrations were measured by a gas chromatograph (GC-2010plusAT, Shimadzu, Japan), equipped with a flame ionization detector with column specifications and procedure parameters described previously [24].

### 2.5. Microbial Community and Bioinformatics Analysis

Samples were obtained from the PBIOA, PSA, PS, BIOmin, BIOmed, and BIOmax reactors at the end of the anaerobic digestion (60 days) for high throughput 16S rRNA gene sequencing analysis. Extraction of DNA was performed with the NucleoBond^®^ Soil kit (Macherey Nagel GmbH & Co. KG, Düren, Germany) as per the instructions provided by the manufacturer. Multiplexed amplicon sequencing analysis of the bacterial 16S rRNA gene was performed as described previously [25]. Briefly, the DNA extracts were used as templates in a two-step polymerase chain reaction (PCR) process, which involved: (i) an initial amplification of the target sequence and (ii) the sample-wise indexing of the PCR products using indexed primers at their 5′ ends [26]. The 515f-806r set of primers (515f 5′-GTGYCAGCMGCCGCGGTAA-3′, 806r 5′-GTGYCAGCMGCCGCGGTAA-3′) were used in the 1st step [27,28], while the index containing constructs (index parted by a 9-bp sample-specific barcode, followed by a 2-bp linker) of the forward primer were used in the 2nd step were used for the 1st step PCR products. PCR reaction mixtures, thermal cycling conditions, and the determination method of the PCR product concentrations are described in our previous study [29]. Before sequencing, the sample-wise indexed products were pooled in equimolar amounts and purified using the Agencourt AMPure XP PCR purification kit (Beckman Coulter, Brea, CA, USA). Libraries were prepared from the multiplexed PCR products with the TruSeq DNA PCR-Free kit (Illumina, San Diego, CA, USA), and were sequenced using an Illumina HiSeq 2500 sequencer at Admera Health (South Plainfield, NJ, USA). The resulting 250 bp paired-end reads were demultiplexed to their samples of origin and further processed with the dada2 v1.14.1 package [30] of the R v3.6.2 statistical software [31]. A table with the amplicon sequence variant (ASV) composition of the samples was generated after sampling index sequence removal (11 bp upstream of the forward primer site) and sequence quality trimming and control, using the default parameters The sequences were annotated using the Silva v138 16S rRNA gene reference database and off-target sequences were removed from the downstream analysis. The raw sequences are publicly available through the Sequence Read Archive (SRA) of the National Center for Biotechnology Information (NCBI) under the Bioproject number PRJNA974352.

### 2.6. Statistical Analyses

The mean values and standard deviation were calculated from the derived data of analytical methods. The comparison of means was performed using variance analysis with one factor (ANOVA), and the differences in values per pair were evaluated using the LSD post hoc test performed with the IBM SPSS Statistics, v.25. Statistically significant differences were considered when the *p*-value was less than 0.05.

### 2.7. Calculations

The μmax, the maximum specific growth rate, was calculated from the slope of the linear segment of the semi-logarithmic graph of methane production against incubation time [32].

Calculations of *FAN* concentrations were conducted based on the following Equation (1):

(1) $$FAN=\frac{TAN}{1+\frac{10^{-pH^{’}}}{K_{a}}}$$

where *K_a_* is the dissociation constant which at the temperature of 37 °C is equal to 1.29 × 10^−9^ [13].

## 3. Results & Discussion

### 3.1. Anaerobic Digestion

The PSA reactors were operated at a TAN of 4379 mgL^−1^, and no bioaugmented microbial culture was added to these reactors. The PS reactors served as the control group without ammonia toxicity, with a concentration of 1598 mgL^−1^, and were used to measure methane production from the cattle manure substrate. In contrast, the BIOmin, BIOmed, and BIOmax reactors, which received bioaugmented microbial culture, exhibited ammonia concentrations of 4256, 4133, and 4011 mg TAN L^−1^, respectively.

**Figure 1:**
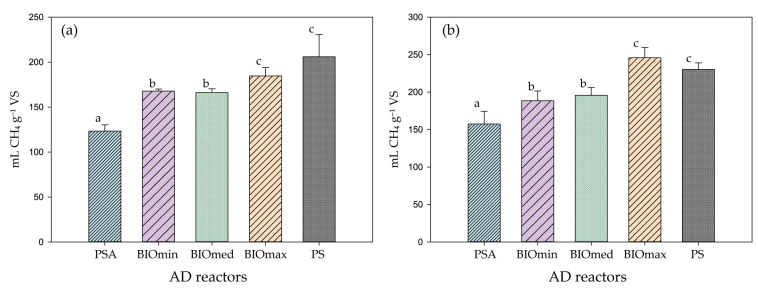
Mean methane production (bars) and standard deviation (error bars) of the bioaugmented reactors BIOmin, BIOmed, and BIOmax and PSA and PS reactors after 30 (**a**) and 60 (**b**) days of continuous anaerobic digestion (AD). Distinct statistical groups (with *p*  <  0.05) between the various cultures are denoted by unique letters above the bars.

Methane production from IN reactors was subtracted from the production of reactors to which inoculum was added. Moreover, the methane production of PBIOA reactors was proportionally subtracted from the production of BIOmin, BIOmed, and BIOmax reactors. The methane potential after 30 and 60 days of anaerobic digestion from all treatments is presented in Figure 1. A typical 30 days period [33] was used to monitor the methane production but the total period of anaerobic digestion experiments was 60 days, as suggested by previous researchers [34]. During the 30-day period, the BIOmax reactors (196.18 ± 9.26 mL CH_4_ g^−1^ VS), which had a high ammonia concentration and a bioaugmented population, did not exhibit a significant statistical difference compared to the PS reactors (205.94 ± 24.75 mL CH_4_ g^−1^ VS), which were used to measure the methane potential of the substrate (cattle manure). The PSA reactors (123.42 ± 6.92 mL CH_4_ g^−1^ VS), where the AD of the substrate with the addition of ammonia was performed, showed noticeably lower methane production compared to the other treatments.

Methane production in the BIO reactors was significantly higher than in the PSA reactors, indicating that bioaugmentation was achieved regardless of the critical biomass introduced into the batch reactors. However, only the BIOmax reactors managed to reach the performance of the PS reactors, and BIOmin and BIOmax methane production was significantly lower in comparison with the PS reactor’s methane production. The BIOmin (167.84 ± 2.30 mL CH_4_ g^−1^ VS) and BIOmed (166.17 ± 4.21 mL CH_4_ g^−1^ VS) reactors did not exhibit a statistically significant difference between them but showed a significant difference compared to the BIOmax reactors.

Notably, even after 60 days of PSA reactors (157.46 ± 17.06 mL CH_4_ g^−1^ VS), which operated under ammonia toxicity, the microbial population was unable to adapt and approach the methane production achieved by the BIOmin (188.52 ± 13.01 mL CH_4_ g^−1^ VS), BIOmed (195.75 ± 10.36 mL CH_4_ g^−1^ VS), BIOmax (245.88 ± 13.60 mL CH_4_ g^−1^ VS) and PS reactors (230.26 ± 8.78 mL CH_4_ g^−1^ VS). In this case, it was confirmed that direct exposure of microbial populations to high levels of ammonia did not lead to adaptation (even after 60 days) as it was also suggested by previous researchers [35]. The statistically significant differences remained the same at 30 and 60 days, as shown in Figure 1. It is important that also after 60 days, there was no statistically significant difference between BIOmax and PS reactors (operated under non-toxic ammonia conditions). The BIOmax reactors exhibited the highest methane production rates under high ammonia concentrations after 60 days of AD.

**Figure 2:**
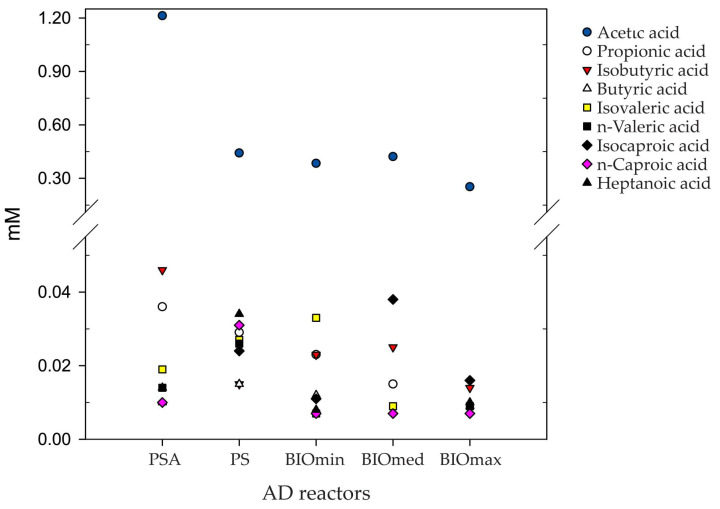
Volatile fatty acids (VFA) concentrations in mMolarity (mM) from samples obtained from all the anaerobic digestion (AD) reactors.

In Figure 2, the profile of volatile fatty acids from all the tested reactors after 60 days is presented. The results are consistent with the methane production results presented in Figure 1. Specifically, where methane production was high, the concentration of volatile fatty acids was low. In the PSA treatment where methane production was low and there was ammonia toxicity, acetic acid was significantly higher compared to other treatments. The increased concentration of acetic acid indicates the lower activity of methane-producing microorganisms that utilize acetic acid, which was caused by high ammonia concentrations. It is noteworthy that propionic acid was not detected in the BIOmax reactors, while it had a higher concentration in the PSA reactors. Propionic acid is considered by many researchers to be the most suitable indicator of the instability of anaerobic digestion [36]. Therefore, in terms of VFA analysis, stable operation of the AD was achieved in all BIO reactors, regardless of the high ammonia concentration. In contrast, the PSA reactors exhibited reduced methane production and demonstrated symptoms of ammonia toxicity.

**Figure 3:**
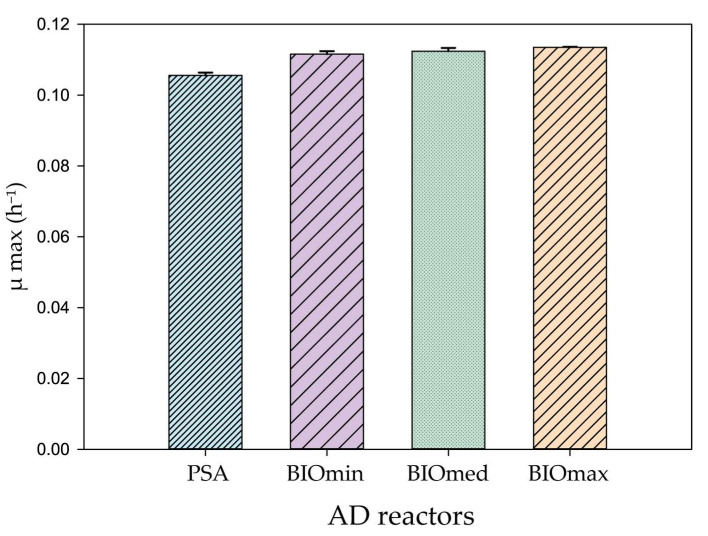
Maximum specific growth rate μmax for the BIO and PSA anaerobic digestion (AD) reactors. Standard deviation is derived from the three replicates performed in each treatment and are presented as error bars.

The maximum specific growth rate was calculated from the maximum values of the exponential phase, as mentioned in Section 2.7, and is presented in Figure 3. The highest μ max was calculated for the BIOmax reactors, and it was 0.1135 ± 0.00014 h^−1^, with a doubling time of 6.1 h. The lowest μ max was of the PSA reactors, which was 0.1056 ± 0.00077 h^−1^, with a doubling time of 6.5 h. Moreover, the other two BIO treatments also had high μ max values, and therefore bioaugmentation was successful in all BIO reactors.

Based on these results, introducing a critical biomass of 65.62% (BIOmax) of the working reactor volume will be preferable for bioaugmentation to alleviate ammonia inhibition in experiments with continuous reactors.

### 3.2. Effect of Ammonia and Bioaugmentation on the Microbial Community of Anaerobic Reactors

Samples were collected from the BIOmin, BIOmed, BIOmax, PS, PSA, and PBIOA reactors for microbial analysis. The analysis was performed with the 515F/806R primer set targeting the V4 region of the 16S rRNA gene of most prokaryotes [26,27] for gaining insight into their community composition. The obtained sequences from reactor samples were predominantly assigned to bacteria, with bacterial ASVs comprising 86.95% of the total, while archaeal ASVs accounted for 11.57%. The results of the microbial analysis for the bacterial and archaeal community structures are presented below.

#### 3.2.1. Bacterial Community Composition

**Figure 4:**
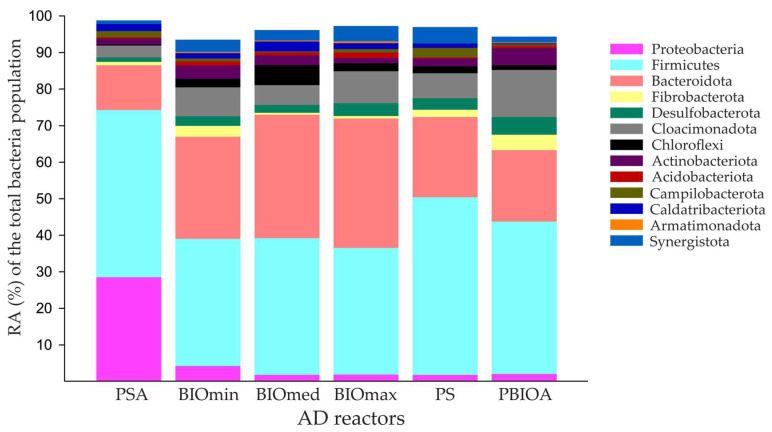
Differences in the relative abundance (%RA) of bacteria at the phylum level based on the overall bacterial population detected in the different anaerobic digestion (AD) reactors.

The most dominant bacterial phyla that were detected in the microbial community of the reactors are presented in Figure 4. From the bacterial sequencing data analysis, most of the sequences were classified into two phyla: Firmicutes 36.1% and Bacteroidota 21.48% with a similar overview of bacterial communities reported by previous researchers [10,14]. The next two phyla with significant representation of their ASVs (6–7%) were Cloacimonadota and Proteobacteria. Cloacimonadota had a high relative abundance in the bioaugmentation culture and in the BIOmax reactors and a low relative abundance in the PSA reactors, which may indicate a sensitivity of these bacteria to a direct increase of ammonia concentrations. Cloacimonadota are known to thrive in anaerobic digesters with lipid-rich waste [37] and as acetogenic microorganisms that can convert amino acids into 2-oxoacids, which can be utilized for carbon and energy [38]. In the case of PSA treatment, the phylum of Proteobacteria was relatively more abundant compared to all other treatments, but also with a significant representation of the other reactor bacterial communities. Proteobacteria are of paramount importance for AD because are known for their ability to utilize glucose, propionate, butyrate, and acetate [39]. The most dominant class of Proteobacteria was γ-Proteobacteria (77.59%) with α-Proteobacteria (22.41%) coming second. The phylum Synergistota was significantly present in the BIO and the PS reactors. All the ASVs assigned to Synergistota belonged to the order Synergistales, which includes some members proposed to be putative syntrophic acetate-oxidizing bacteria [40]. The lowest relative abundance of Synergistota was found at the PSA reactors where ammonia was increased abruptly at toxic levels by direct addition of NH_4_Cl solution without bioaugmented culture inoculation.

#### 3.2.2. Archaeal Community Composition

The archaeal phylum with the highest relative abundance was Halobacterota, accounting for 81.93% of the total archaeal ASVs, followed by Crenarchaeota with 15.21%. The remaining archaeal phyla detected by DNA analysis were Thermoplasmatota and Euryarchaeota, representing 12.02% and 3.45% of the total archaeal ASV population, respectively. In the bioaugmentation culture (PBIOA reactors), the relative abundance of Archaea was higher and reached 21.79% of the total prokaryotic population. Moreover, BIOmin (18.10%), BIOmed (6.93%), and BIOmax (14.84%) reactors had a higher percentage of the mean relative abundance of Archaea compared to the PSA (5.16%) reactors, which can be explained by the introduced bioaugmented culture in the BIO reactors.

**Figure 5:**
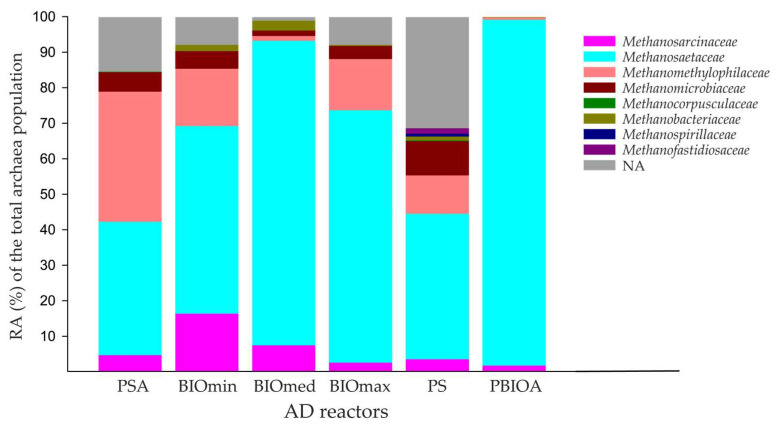
Differences in the relative abundance (%RA) of Archaea at the family level based on the overall Archaea population detected in the different anaerobic digestion (AD) reactors.

The relative abundance of archaeal families among the overall archaeal populations detected in samples of the AD reactors are presented in Figure 5. Members of the *Methanosaetaceae* family were dominant in terms of relative abundance in all treatments. In accordance with a previous study, where *Methanosaetaceae* family members were found to have a high relative abundance and to be more actively involved in methanogenesis under ammonia-associated inhibition [41]. Specifically, members of this family were the most abundant in the bioaugmentation culture (PBIOA) at 97.40%, as well as in the bioaugmentation reactors of BIOmed, BIOmin, and BIOmax, with a relative abundance among Archaea of 85.74%, 52.86%, and 71.09%, respectively. The *Methanosaetaceae* family was represented only by the genus *Methanosaeta*, which is generally known as obligate acetoclastic methanogen [12,42]. This result is in accordance with the findings of a previous study where, in all AD reactors facing ammonia toxicity, the genus *Methanosaeta* was identified as one of the two most dominant genera, following the use of various bioaugmentation cultures [12]. *Methanosarcinaceae* family members were detected in all reactors. Even though high relative abundances were observed in the BIOmin and BIOmed reactors, there were no statistically significant differences between the other examined reactors. A significant family in terms of relative abundance was *Methanosarcinaceae*, which was detected in all AD reactors. A prevalent member of *Methanosarcinaceae* was the genus *Methanosarcina*, which are known to grow on several simple carbon compounds using the main three methanogenic pathways: acetoclastic (acetate), hydrogenotrophic (CO_2_ + H_2_), and methylotrophic (methanol, methylamines, methylsulfides) [43]. *Methanosarcinaceae* members were often observed in manure-fed reactors and at inhibitory concentration levels of ammonia [44], and their increased adaptation has been associated with the specific cell morphology of the *Methanosarcina* genus which have a high volume-to-surface ratio and are able to create multiple cells structures (clusters) [45].

In the PS reactors, *Methanosaetaceae* and *Methanomethylophilaceae* were the most dominant among families (Figure 5). However, in the PSA reactors, where the only difference with PS was the presence of ammonia at inhibitory concentrations, the relative abundance of *Methanosaetaceae* family members remained almost unchanged, while the abundance of *Methanomethylophilaceae* significantly increased from 10.82% to 36.67% of the total Archaea. The high tolerance of *Methanomethylophilaceae* to ammonia and their increased activity under toxic ammonia concentrations were also reported before in continuously stirred-tank reactors (CSTR) [13]. Members of the *Methanospirillaceae* family (hydrogenotrophic) were likely present only in the initial inoculum used for the anaerobic digestion (AD) of all the reactors, as they were detected solely in the PS reactors that were not exposed to toxic ammonia concentrations. This result is in accordance with a previous study on the effects of ammonia toxicity on syntrophic propionate oxidation in anaerobic digester sludge where hydrogenotrophic *Methanospirillaceae* were moderately and severely inhibited at a concentration of 3 g L^−1^ of ammonium nitrogen [41]. Furthermore, the presence of high ammonia concentrations in the anaerobic digestion reactors had a notable impact on the populations of *Methanofastidiosaceae*, a family known for methane production through the H_2_-utilizing methylotrophic pathway [46]. These *Methanofastidiosaceae* members were exclusively detected in the PS reactors, suggesting that their presence resulted from the initial inoculum used for anaerobic digestion. This finding strongly indicates the influence of elevated ammonia levels on their populations.

In general, the relative abundance of acetoclastic Archaea (*Methanosaeta*, *Methanosarcina*) showed a statistically significant difference compared to hydrogenotrophic and methylotrophic Archaea in all other treatments. However, *Methanosarcina* has been reported to potentially act as a hydrogenotroph under high concentrations of ammonia, thereby probably increasing the activity levels of the specific methanogenic pathway [47,48]. Among the hydrogenotrophic methanogens, members of the *Methanomicrobiaceae*, *Methanobacteriaceae*, *Methanocorpusculaceae*, and *Methanospirillaceae* families were detected in the AD reactors. *Methanomicrobiaceae* exhibited the highest relative abundance in the PS, PSA, BIOmin, and BIOmax treatments, while *Methanobacteriaceae* was the dominant hydrogenotroph in BIOmed reactors. In the case of methylotrophic methanogens belonging to the order Methanomassiliicoccales, the family *Methanomethylophilaceae* was identified as the most dominant in terms of relative abundance among methylotrophic Archaea. According to microbial analysis, the pathway of acetoclastic methanogenesis was likely the dominant for the observed methane production. However, the highest methane production was observed in the BIOmax reactor among those with induced ammonia toxicity. DNA analysis revealed a significant presence of hydrogenotrophic and methylotrophic methanogens in this reactor. Therefore, the ratio between the different types of active methanogens in the AD reactors, particularly those with high concentrations of ammonia and supplemented with a bioaugmentation culture, may be the underlying reason for the high methane production.

## 4. Conclusions

In this study, bioaugmentation successfully addressed ammonia inhibition on methane production in anaerobic reactors. The BIOmin and BIOmed reactors exhibited a significant increase in methane production compared to the control group of PSA reactors with the same ammonia concentration. However, only the BIOmax reactors reached the mean methane production level of the PS reactors (without ammonia toxicity) within 30 days of anaerobic digestion and surpassed it after 60 days. Therefore, the results of this study demonstrate that the critical biomass of the bioaugmented culture to alleviate inhibition due to high ammonia concentrations should be injected at 65.62% of the working reactor volume in a single application. DNA analysis revealed a dominance of acetoclastic methanogens from the *Methanosaeta* genus in all bioaugmented reactors and cultures. Additionally, methylotrophic and hydrogenotrophic methanogens were present in all reactors, indicating the operation of these methanogens (pathways) during AD.

## Figures and Tables

**Table 1:** Characteristics of inoculum used for the anaerobic digestion (AD), inoculum for the creation of the bioaugmented culture, bioaugmented culture, and cattle manure. Values after the ± symbols stand for standard deviation.

Parameter (Unit)	Inoculum for AD	Inoculum for Bioaugmentation Culture	Bioaugmented Culture	Cattle Manure
Total Solids–TS (g L^−1^)	67.10 ± 0.35	65.2 ± 0.34	37.28 ± 0.84	66.6 ± 0.42
Volatile Solids–VS (g L^−1^)	52.52 ± 0.42	50.48 ± 0.45	30.85 ± 0.93	56.05 ± 0.54
Total Kjeldahl Nitrogen–TKN (g L^−1^)	4.06 ± 0.34	4.00 ± 0.28	5.45 ± 0.69	3.60 ± 0.58
Total Ammonia Nitrogen–TAN (g L^−1^)	2.03 ± 0.18	2.53 ± 0.25	5.20 ± 0.19	1.57 ± 0.12
pH	7.8 ± 0.05	7.7 ± 0.05	7.8 ± 0.03	7.60 ± 0.04
Total Volatile Fatty Acids–VFAs (g L^−1^)	0.10 ± 0.003	0.10 ± 0.001	0.12 ± 0.001	11.61 ± 0.15

**Table 2:** Sets (triplicates) of anaerobic digestion (AD) reactors used for the experimental procedure and their contents (nominal values).

AD Reactors	Inoculum for AD (g)	Cattle Manure (g)	Ammonia (NH_4_Cl 50 g L^−1^) (g)	Bioaugmented Culture (g)	Water (g)	Total (g)
PSA	112	18	8.90	-	21.10	160
PS	112	18	-	-	30.00	160
IN	112	-	-	-	48.00	160
PBIOA	-	-	13.50	10.09	136.41	160
BIOmin	112	18	8.50	6.96	14.54	160
BIOmed	112	18	8.10	13.90	8.00	160
BIOmax	112	18	7.70	20.85	1.45	160

## Data Availability

Raw sequences are publicly available at the National Center for Biotechnology Information (NCBI) under the Bioproject number PRJNA974352, https://www.ncbi.nlm.nih.gov/search/all/?term=PRJNA974352 (accessed on 26 June 2023).
